# Validating a standardised test battery for synesthesia: Does the Synesthesia Battery reliably detect synesthesia?

**DOI:** 10.1016/j.concog.2015.02.001

**Published:** 2015-02-28

**Authors:** D.A. Carmichael, M.P. Down, R.C. Shillcock, D.M. Eagleman, J. Simner

**Affiliations:** a Dept. of Psychology, University of Edinburgh, 7 George Square, EH8 9JZ, UK; b Institute for Adaptive & Neural Computation, University of Edinburgh, 10 Crichton Street, EH8 9AB, UK; c Division of Psychiatry, University of Edinburgh, Royal Edinburgh Hospital, EH10 5HF, UK; d Department of Neuroscience, Baylor College of Medicine, Houston, TX, USA; e School of Psychology, University of Sussex, Falmer BN1 9QH, UK

**Keywords:** Synesthesia, Validation, Test, Assessment, Consistency, Prevalence, Grapheme-colour

## Abstract

Synesthesia is a neurological condition that gives rise to unusual secondary sensations (e.g., reading letters might trigger the experience of colour). Testing the consistency of these sensations over long time intervals is the behavioural gold standard assessment for detecting synesthesia (e.g., Simner, Mulvenna et al., 2006). In 2007 however, Eagleman and colleagues presented an online ‘Synesthesia Battery’ of tests aimed at identifying synesthesia by assessing consistency but within a single test session. This battery has been widely used but has never been previously validated against conventional long-term retesting, and with a randomly recruited sample from the general population. We recruited 2847 participants to complete The Synesthesia Battery and found the prevalence of grapheme-colour synesthesia in the general population to be 1.2%. This prevalence was in line with previous conventional prevalence estimates based on conventional long-term testing (e.g., Simner, Mulvenna et al., 2006). This reproduction of similar prevalence rates suggests that the Synesthesia Battery is indeed a valid methodology for assessing synesthesia.

## 1 Introduction

*Synesthesia* is an inherited condition in which everyday stimuli trigger unusual secondary sensations. For example, synesthetes listening to music might see colours in addition to hearing sound (Ward, Huckstep, & Tsakanikos, 2006). One particularly well-studied variant is *grapheme-colour synesthesia*, in which synesthetes experience colours when reading, hearing or thinking about letters and/or digits (e.g., Simner, Glover, & Mowat, 2006). Despite being first reported over two hundred years ago (by Sachs, 1812; see Jewanski, Day, & Ward, 2009) synesthesia was initially an under-researched and poorly-understood area of human experience until the last decades of the 20th century. A significant factor in the elevation of synesthesia as a tractable topic was the realisation – and subsequent empirical confirmation – that synesthetes’ experiences could be verified behaviourally by the fact that they remain conspicuously stable over time (Baron-Cohen, Wyke, & Binnie, 1987; Jordan, 1917). Specifically, synesthetes tend to be highly consistent when reporting their synesthetic sensations for any given stimulus. For example, if the letter J triggers the colour pale blue for a given synesthete, she will tend to repeat that J is pale blue (not green, not yellow, etc.) when repeatedly tested over days, months and even years. Indeed, one study was able to show that synesthetic sensations had remained consistent over at least three decades (Simner & Logie, 2008).

This stability of responses over time is considered one of the central features of synesthesia and is routinely verified in almost every publication on the subject (e.g., Asher, Aitken, Farooqi, Kurmani, & Baron-Cohen, 2006; Baron-Cohen, Burt, Smith-Laittan, Harrison, & Bolton, 1996; Rich, Bradshaw, & Mattingley, 2005; Ward & Simner, 2003; but see Simner, 2012). In other words, while a wide range of behavioural approaches have been employed to assess the nature of the synesthetic experience, experimental methodologies aiming to *validate* synesthesia have almost exclusively focussed on the feature of consistency. Hence, researchers selecting synesthete participants for study first verify the genuineness of each case by requiring their synesthetes to demonstrate high levels of consistency over time compared to non-synesthete controls (e.g., Asher et al., 2006; Baron-Cohen et al., 1996; Simner et al., 2006). Controls are tested on analogous associations (i.e., they invent colours for the 26 letters, say, and then attempt to recall these colour associations later) and typically perform significantly worse than synesthetes.

Although more than a hundred contemporary studies rely on this test of consistency for genuineness, the particular instantiation of the test has varied widely. For example, a wide range of methods have been used to elicit synesthetic colours: participants have indicated these by either giving verbal descriptions (e.g., Ward, Simner, & Auyeung, 2005), written descriptions (Simner, Glover et al., 2006), using Pantone© swatch colour charts (Asher et al., 2006), electronic colour charts (Simner, Harrold, Creed, Monro, & Foulkes, 2009) or even computerised colour pickers offering extensive palettes of >16 million colours (e.g., Simner & Ludwig, 2012). In this way, synesthesia research has used varying methods, which in turn might raise difficulties for researchers when trying to meaningfully compare data.

Despite this superficial variability however, the test of genuineness has nonetheless tended to rely on one key shared feature: synesthetes must outperform controls over fairly lengthy re-test intervals. Consider, for example, the most widely cited large-scale screening for synesthesia (Simner, Mulvenna et al., 2006) in which a large sample of participants were opportunistically recruited from the communities of Edinburgh and Glasgow Universities, and individually assessed for synesthesia. Participants first indicated by questionnaire whether they believed they experienced synesthesia, and those who reported in the affirmative were asked to provide their synesthetic associations (e.g., the colours of letters). These participants were then retraced after considerable time had passed (on average 6.0 months) and were asked in a surprise retest to re-state their associations. A group of controls without synesthesia performed an analogous task but were re-tested after only two weeks. Synesthetes were able to significantly out-perform controls even though much time had passed and the deck was effectively stacked against them. Methodologies such as this allow confident detection of genuine synesthetes because the surprise retest over lengthy intervals places the performance of synesthetes beyond the usual abilities of the average person. The drawback to this methodology, however, is that the task is extremely time-intensive to perform, and risks a high drop-out rate if synesthetes become untraceable at retest.

Perhaps for this reason, one of the most important developments in the methodology of synesthesia validation came with the introduction in 2007 of an alternative version of the test of genuineness. Eagleman and colleagues produced the *Synesthesia Battery*, a toolbox of online tests which provides a standardised set of questions, tests and quantitative scores to assess a range of synesthesias (Eagleman, Kagan, Nelson, Sagaram, & Sarma, 2007). This battery is again based on internal consistency in that synesthetes are validated by high consistency within their own synesthetic associations, stated repeatedly. However, consistency is measured within a single test session lasting only approximately 10 min. Specifically, synesthetes log on to the testing site (www.synesthete.org) and specify which form(s) of synesthesia they experience. The testing platform then presents their triggering stimuli (e.g., the 26 letters) one by one in randomized order, and participants are required to select their synesthetic colour for each trigger. Each stimulus (e.g., letter) is presented three times each, and a score is generated to quantify the consistency of participant’s responses (e.g., did the participant choose the same/similar colours each of the three times she saw a particular letter?) This score represents the geometric distance in RGB (red, green, blue) colour space, where R, G, and B values are all normalised to lie between 0 and 1. If the mean overall score of colour-distance is less than 1, the participant is classified as a synesthete; if the score is 1 or higher, the degree of inconsistency classifies the participant as a non-synesthete. However, it remains an open question whether this limited retest interval is sufficient to truly distinguish synesthetes from non-synesthetes.

In the current study we assessed the validity of the Synesthesia Battery by using it to test almost 3000 randomly sampled subjects for grapheme-colour synesthesia. Our aim was to establish the prevalence of grapheme-colour synesthesia by this method. This will allow us to evaluate the Synesthesia Battery by comparing this prevalence – obtained by assessments within in a single test session – to the most widely accepted previous estimate of the prevalence of grapheme-colour synesthesia based on the standard *longitudinal* test–retest method (Simner, Mulvenna et al., 2006). If the Synesthesia Battery is just as effective a method for detecting synesthesia as the more standard long-term retest method, we anticipate an equivalent prevalence of grapheme-colour synesthesia across both methods. In carrying out our study, we chose to evaluate grapheme-colour synesthesia in particular for several reasons: it is one of the most common forms of synesthesia (Simner, Mulvenna et al., 2006), it is particularly well-understood in behavioural terms, it lends itself readily to online testing, and those who experience it typically demonstrate the high levels of consistency expected from synesthetes (compared to other variants, whose more complex concurrents may make them more difficult to assess via consistency alone; see Simner, Gäartner, & Taylor, 2011 for discussion). It was not our intention to change or try to improve upon the method made available by Eagleman et al. at www.synesthete.org. Rather, we attempted to simply replicate their test and methodology and then evaluate how it performs in comparison to a conventional longitudinal test–retest method.

In evaluating the Synesthesia Battery, our data will also provide an independent test of the prevalence of synesthesia. Our baseline study – the widely cited prevalence study of Simner, Mulvenna et al. (2006) – found the prevalence of grapheme-colour synesthesia to be 1.4% (for synesthetes with both coloured letters *and* numbers) or 2% (for synesthetes with either coloured letters *or* numbers). This study was based on a sample of 500 individuals, and the prevalence rate it generated was subsequently verified by a secondary method testing a further 1190 individuals (see Section 4 for details of this second method).

Two previous studies have also aimed to validate aspects of the Synesthesia Battery (Eagleman et al., 2007; Rothen, Seth, Witzel, & Ward, 2013). Both studies used self-reported synesthetes who had self-referred for study, in comparison to a group of controls declaring they were non-synesthetes. it is important to highlight the difference between *self-referred* and *self-reported* synesthetes. Self-reported synesthetes are any individuals who claim they have synesthesia. Self-referred synesthetes are those who have additionally made the effort to contact a university researcher to volunteer to take part in synesthesia studies. All synesthetes tested by Eagleman, Rothen and colleagues were not only self-declared, but also, importantly, self-referred. In comparison, none of the synesthetes tested here are self-referred. Instead, our approach is to screen the general population (some of whom at a certain point during our test, will self-report having synesthesia when asked, but will not be self-referred). There are likely to be significant differences between our own synesthetes, and the self-referred synesthetes of Eagleman, Rothen and colleagues. These latter synesthetes not only know they have coloured letters, but also know this is called synesthesia, and furthermore, they have made the effort to contact a university researcher to volunteer to take part in synesthesia studies. They therefore have an understanding of synesthesia and judge that the extent of their synesthesia is worthy of study by researchers. In other words, it is at least possible that self-referrers have relatively ‘strong’ (or noticeable, or attention-catching) synesthesia in some way and may not be entirely representative of the population of synesthetes at large.

In summary, because of the sampling methods of the two previous validations (Eagleman et al., 2007; Rothen et al., 2013) their participant groups (synesthetes versus controls) may have had diametrically opposing synesthesia characteristics, which might have therefore made them relatively easy to distinguish between. Indeed, both Eagleman et al. (2007) and Rothen et al. (2013) obtained a bimodal distribution of scores when assessing the consistency of grapheme-colour associations of their self-referred synesthetes compared to controls. Here however we individually assess a *randomly recruited* sample of subjects, allowing our own study to extend the previous findings of Eagleman et al. and Rothen et al. and establish how the Synesthete Battery performs when a distinction between synesthetes and non-synesthetes is perhaps more difficult to achieve. Put differently, by testing a random sample of the population, we expect to capture a broader, more representative range of synesthetic experiences, and we are evaluating how the Synesthete Battery performs under these conditions. In addition, our study will provide the largest estimate of the prevalence of grapheme-colour synesthetes to date, with almost 3000 randomly sampled members of the general population.^1^

Finally, our study also investigates a second aspect of the Synesthesia Battery. After the single session test of consistency of coloured graphemes, participants next immediately perform a second test of synesthesia: a *speeded congruency verification task* (Eagleman et al., 2007). In this, participants are presented with individual graphemes, but this time the graphemes are coloured either to match the participant’s earlier colour selection (congruent), or to be a different colour (incongruent). Participants must simply answer whether the letter-colour pairing matched their previous choices or not, and their accuracy and speed is measured. Eagleman et al. (2007) report that synesthetes tend to score 90% or higher with a mean RT of 0.64 ± 0.78 s, while non-synesthetes score below 90% with mean RT of 0.91 ± 0.87 s (Eagleman et al., 2007). We will examine the sensitivity of this type of test to determine whether it too has the diagnostic capability to distinguish between participants who score below the consistency threshold score of <1 (i.e., synesthetes) and those who do not. In other words, where Eagleman et al. (2007) compared self-referred synesthetes with non-synesthete controls, we will extend this type of test to assess (a) randomly sampled participants, who are (b) self-declared synesthetes, but who are not self-referred synesthetes, and who are also (c) either verified as genuine versus non-genuine. As such, we are evaluating whether this type of speed-congruency test still holds up in what is likely to be a more sensitive comparison.

## 2 Methods

### 2.1 Participants

Two thousand eight hundred and forty-seven participants took part in our study (1317 male, 1530 female; mean age 28.6, range 16–90, S.D. 14.3). We had additionally tested 32 further subjects who completed our study but had entered an obviously false date of birth (e.g., 2013). These subjects did not enter our analysis, which was therefore based only on our *N* = 2847.

Participants were recruited as part of a large-scale, centrally co-ordinated undergraduate research project. Every student registered on the 2nd year of the Psychology undergraduate course at the University of Edinburgh acted as a research assistant (RA), and was required to each recruit 8 participants (4 male and 4 female) over 16 years of age. Our student RAs were not allowed to take part in the study themselves. In recruiting our participants, we took a number of steps to ensure as random a sample as possible. First, RAs were instructed not to deliberately seek out, nor to avoid, people they knew to be synesthetes. Furthermore, in order to avoid self-referral biases, RAs were required to pre-select their sample, and then approach participants in a targeted way (rather than send out an advert and accept self-referrals). Indeed, RAs were required to refrain from recruiting participants via any open calls at all, for example, they could not post the testing URL on social media websites or internet forums. Finally, RAs were also instructed not to *a priori* inform participants that the study involved synesthesia. The instructions given by the RAs to prospective participants were uniform, and clearly stated that participants were only allowed to complete the test once; if they had previously been approached to complete the test by someone else, they were to inform the recruiter and not proceed with the test.

Our study was carried out in two waves to maximise participation numbers: 1514 were tested in January 2013, and 1333 were tested in September 2013. Both used identical methods, carried out by two consecutive intakes of 2nd year students. In both rounds, the study was carefully managed and co-ordinated by authors JS and DAC. Data from both rounds are pooled and presented together here.

### 2.2 The online test

The online test consisted of several sections. Participants first provided informed consent via a checkbox and then gave demographic information such as age, sex, handedness and native language. A second section consisted of a health questionnaire not relevant for the current study. (In this, subjects were requested to indicate if they suffered from a range of clinical conditions, and this was for another project to be reported elsewhere.) After this page, our online synesthesia assessment began with our locally stored replica of the Synesthesia Battery. In this replica – as in the original – participants were first asked whether they experienced grapheme-colour synesthesia, with the question “Do numbers or letters cause you to have a colour experience?” This was accompanied by an example, and then an option to accept separately according to whether these colours are triggered automatically by numbers and/or digits. If participants indicated that they saw neither letters nor numbers in colour, they advanced to an early-exit page thanking them for their participation.

**Fig. 1:**
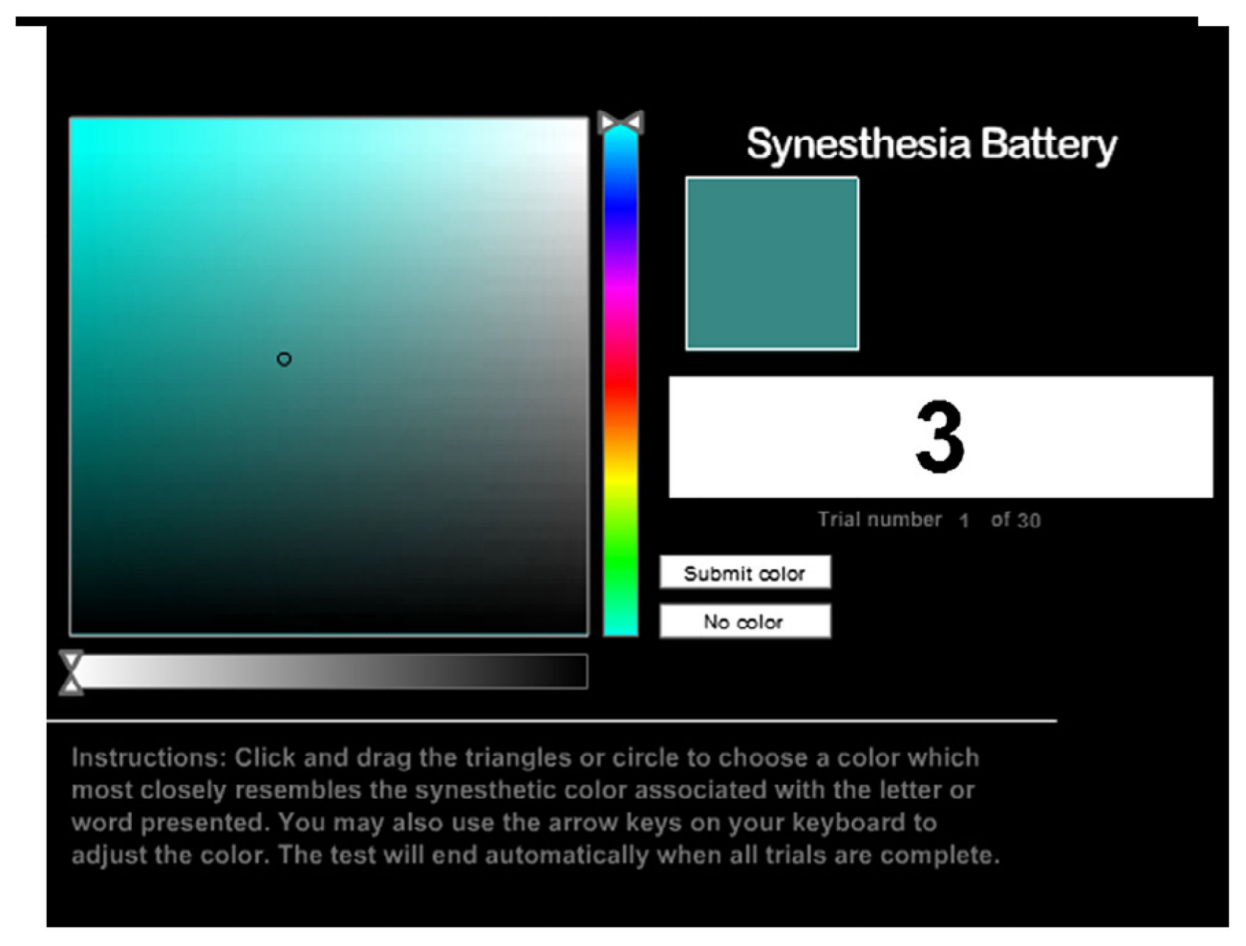
Screenshot from the consistency test.

The rest of the test was completed by participants who answered in the affirmative to having coloured letters/digits. These participants completed two further tests tailored to the particular variant of grapheme-colour synesthesia they had reported (i.e., for either digits, letters, or both). These two tests were a colour consistency test and a speeded congruency task. The colour consistency test was again an identical clone of the consistency test from the Synesthesia Battery (Eagleman et al., 2007). In this, participants were presented with each grapheme (a–z, 0–9) three times in random order (so 30 trials if the subject reported coloured numbers only, 78 trials if letters only, and 108 trials if both letters and numbers). For each trial, participants were required to select the colour that best matched the grapheme presented (see Fig. 1). Selections were made from a palette of 256 × 256 × 256 colours, exactly as in the original Synesthesia Battery. Once their selection was submitted, the screen advanced to show the next grapheme. The colour palette followed an HSL colour model, with colours varying in lightness along the vertical axis and saturation along the horizontal access a separate, horizontal bar allowed hue to be adjusted (see Fig. 1).

**Fig. 2:**
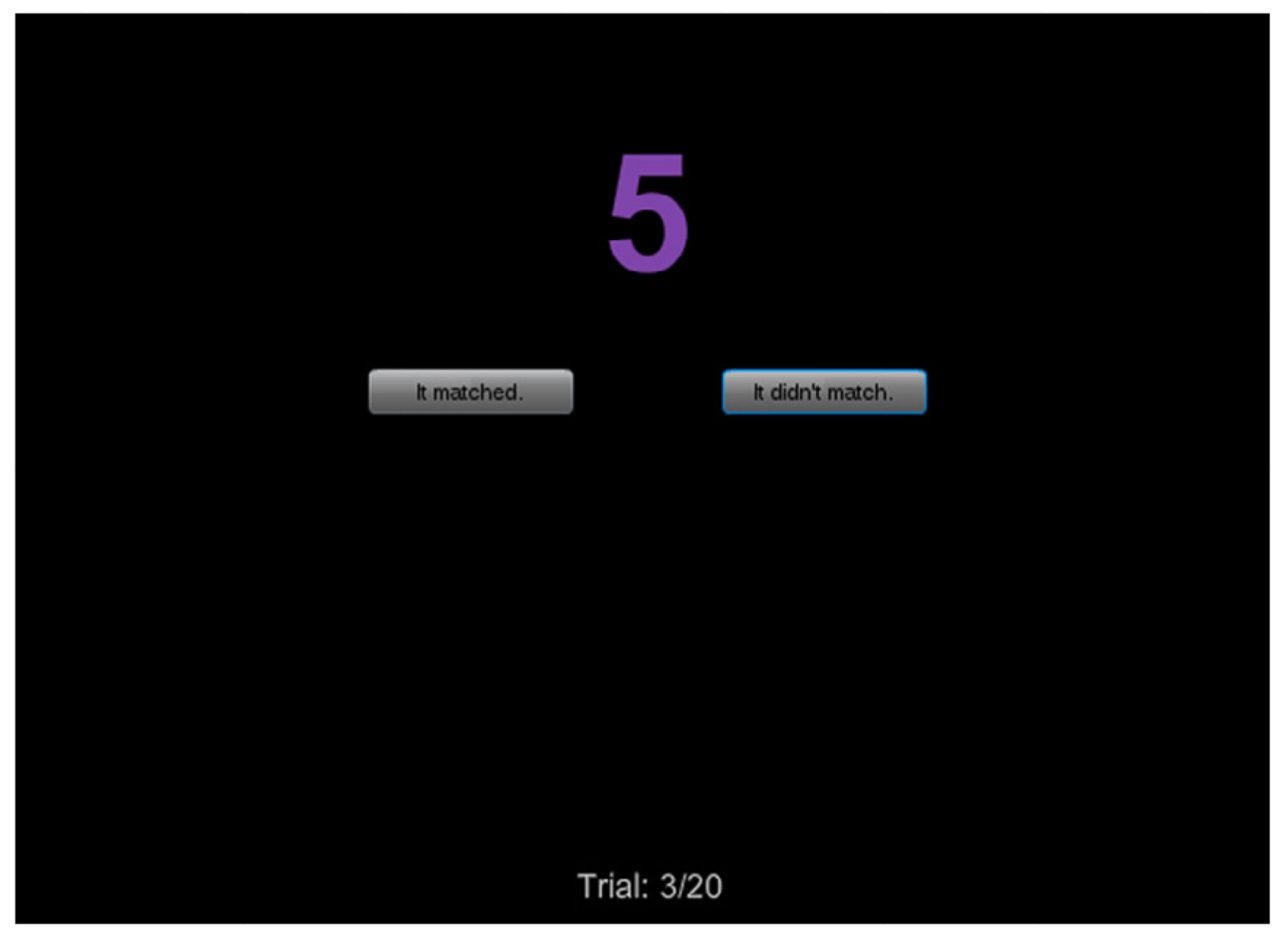
Screenshot from the speeded congruency task.

The colour consistency test was followed by a speeded congruency task. In this section, participants were shown again the graphemes they had just seen in the colour consistency test. This time they saw each relevant grapheme twice, in a random order, each flashed on screen for a maximum of 1 s or until the participant responded (20 trials for just numbers, 52 trials for just letters or 72 trials for both letters and numbers). In 50% of trials, graphemes were coloured congruently with the participant’s earlier specification, and in 50% of trials they were coloured incongruently. Participants were required to indicate by mouse-click on the relevant on-screen button whether the each grapheme they saw either matched or did not match their previous colour pairing (as collected during the consistency test; Fig. 2). Their response mouse-click advanced the test to the next grapheme, and the test continued until all graphemes had been shown.^2^

In summary, the colour consistency test generated a consistency colour-distance score, and the speeded congruency task generated an accuracy score and a reaction time. For full details of website configuration and how the consistency colour-distance score is calculated, see Eagleman et al. (2007).

## 3 Results

In our study, we classified as non-synesthetes all those who were directed to the early-exit page (i.e., those who said they did not experience coloured letters and/or digits) and all those who continued but scored 1 or higher. The remainder were classified as synesthetes (i.e., those who scored <1).

From our sample of 2847 participants, 140 subjects (55 male, 85 female, mean age 23.9, range 16–71, SD 9.5) self-reported grapheme-colour synesthesia, giving a self-reported prevalence of 4.9%. Of those 140 self-reported synesthetes, 34 obtained a colour-distance score of <1 on their consistency test (14 male, 20 female, mean age 24.9, range 17–51, SD 5.8), which is the criterion used by Eagleman et al. (2007) to identify genuine synesthesia. This places the prevalence of genuine grapheme-colour synesthesia at 1.2% and we will return to this prevalence value further below.

**Fig. 3:**
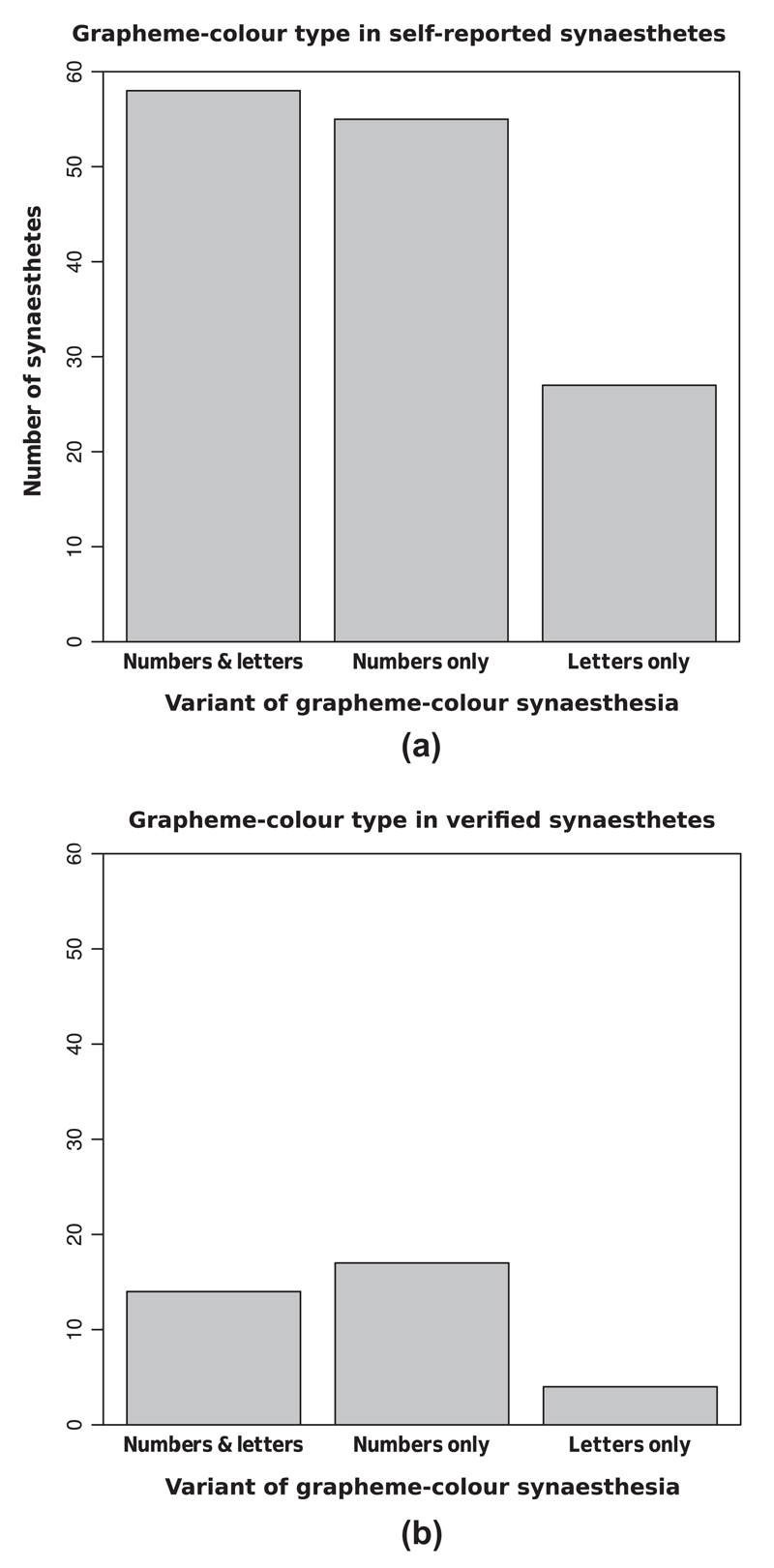
Variants of grapheme-colour synesthesia reported by (a) all 140 participants who self-reported synesthesia, and (b) the 34 participants confirmed as genuine synesthetes (i.e., with colour-distance consistency scores of <1).

Of the 140 self-reported synesthetes, 55 reported experiencing coloured numbers only, 58 reported experiencing both coloured numbers and letters and 27 subjects reported experiencing coloured letters only (see Fig. 3a). Of the 34 participants that scored <1 on the consistency test, 17 experienced coloured numbers only, 14 experienced both coloured numbers and letters and 3 subjects experienced coloured letters only (see Fig. 3b). As an additional check, the colour choices of the participants obtaining a consistency score of <1 were examined individually to allow us to confirm that none of these achieved their superior consistency by entering the same colour for each grapheme, or by entering an obviously non-synesthetic pattern of colours throughout, e.g. red for ‘R’, green for ‘G’ and blue for ‘B’ (following Simner, Mulvenna et al., 2006).

Next we analysed accuracy and RTs in the speeded congruency task. We first divided our sample of self-reported synesthetes into two groups, around to the consistency colour-distance threshold of <1. For clarity, we refer to those who scored <1 as *genuine synesthetes*, and those who score ≥1 we refer to as *malingerers*^3^ (i.e., non-synesthetes who self-reported synesthesia but failed to achieve what is considered a synesthetic score in the colour consistency test). There were 34 genuine synesthetes, as noted above, and 90 malingerers. There were also 16 subjects who reported too few coloured graphemes to generate a consistency colour-distance score at all. Following Eagleman et al., 2007, participants were required to enter a minimum of two valid graphemes to obtain a consistency score, a valid grapheme being defined as one to which the subject entered a colour for all three presentations. These 16 are omitted from all further analyses below. (Finally, we remind the reader that there are no values for participants who declared themselves to be non-synesthetes from the start because these individuals did not progress to the synesthesia assessment.)

**Fig. 4:**
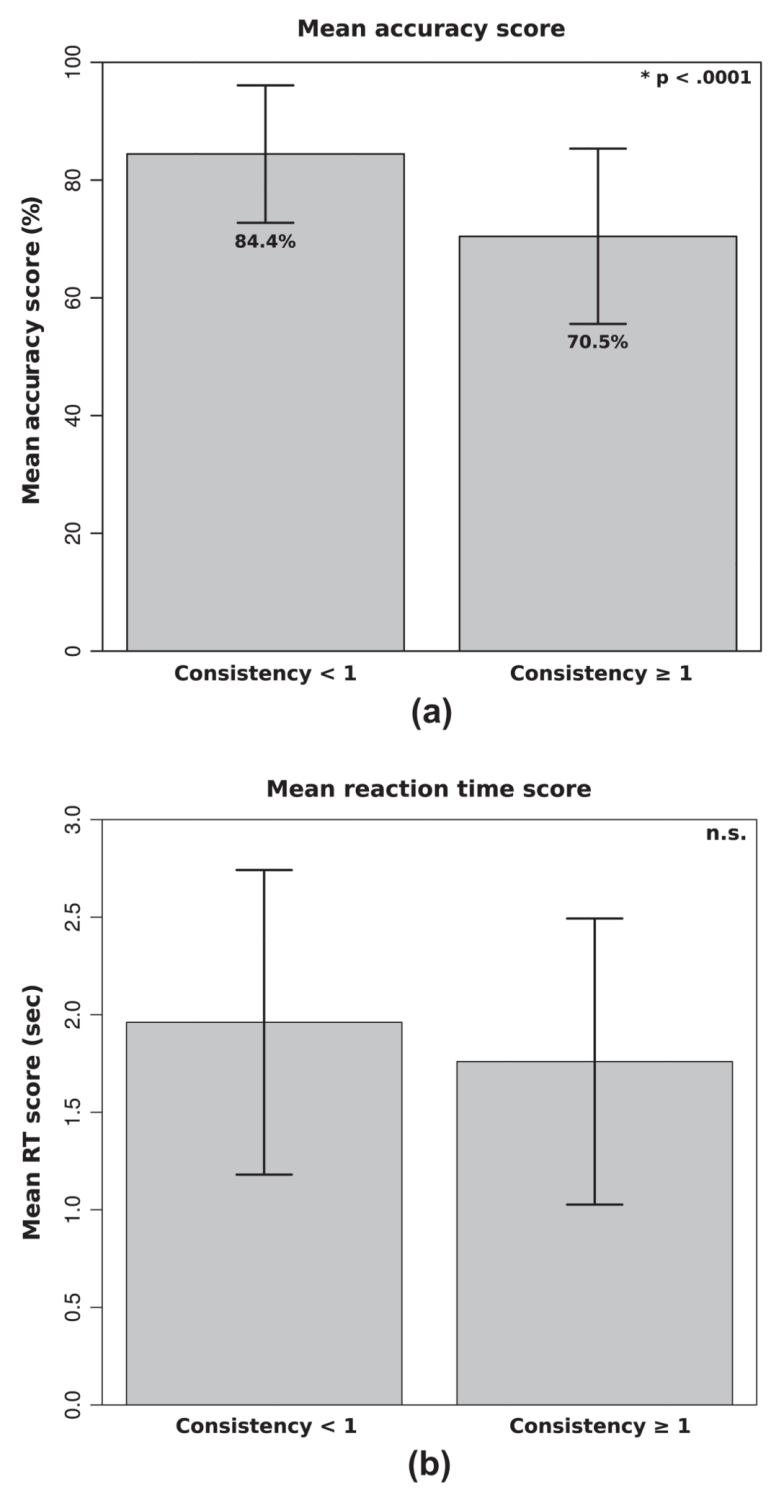
Results from the speeded congruency task showing that (a) genuine synesthetes (indicated as “consistency <1”) were significantly more accurate than malingerers (indicated as “consistency ≥1”) and that (b) there was no significant difference in mean reaction time between these two groups. (Note that there are no values for participants who declared themselves to be non-synesthetes from the start because these individuals did not progress to the synesthesia assessment.)

Using an independent samples *t*-test, we calculated the mean accuracy in the speeded congruency task for each group: genuine synesthetes versus malingerers. This mean was 84.4% (SD = 11.8) for synesthetes and 70.5% (SD = 14.9) for malingerers, and this difference was significant (*t*(122) = 5.45, *p* < .0001, *d* = 1.03). There was no significant difference in mean reaction times between groups (Synesthetes *M* = 1.93 s; SD = 0.77; Malingerers *M* = 1.76 s, SD = 0.73; *t*(122) = 1.13, *p* = .13, *d* = 0.23) see Fig. 4a and b). Mean reaction time was calculated across all trials, irrespective of whether the participant had answered correctly or incorrectly.^4^

In order to investigate whether this non-significant result provided evidence for the null hypothesis we calculated a Bayes factor. By comparing the likelihood of two models (in this case, the null and alternative hypotheses) as a ratio, Bayes factors allow the researcher to evaluate to what extent the data supports the null hypothesis (Rouder, Speckman, Sun, Morey, & Iverson, 2009). Following Jeffreys (1961), a Bayes factor of less than 0.33 provides strong support for the null hypothesis, a Bayes factor of greater than 3 provides support for the alternative hypothesis and values inbetween indicate the data are insensitive and no firm conclusions should be drawn. Using the online calculator provided by Rouder et al. (2009), we calculated a Bayes factor of 1.17, indicating that the data are not sensitive enough to enable a conclusion to be drawn.

**Fig. 5:**
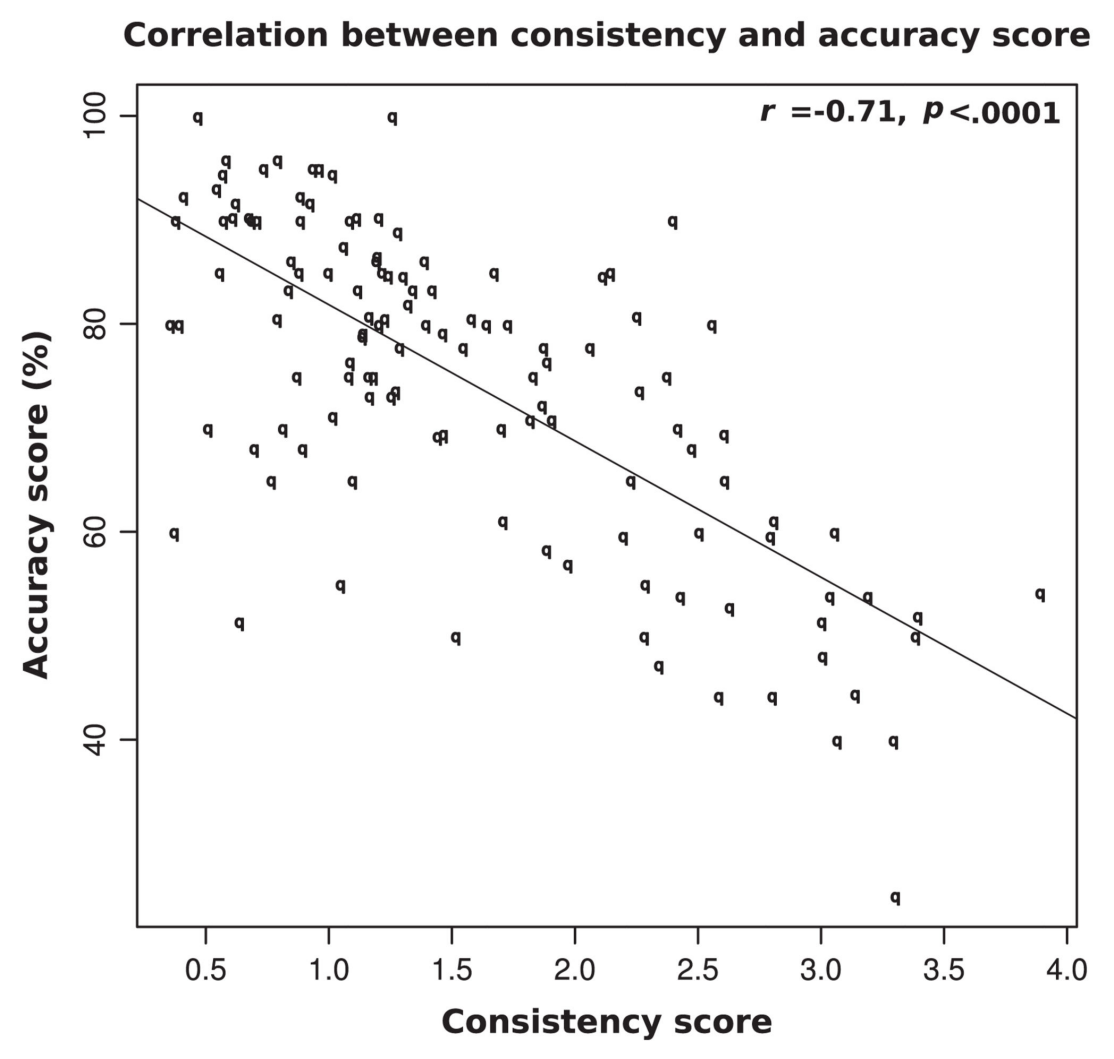
Scatterplot showing a significant association between colour-distance consistency score and accuracy score for self-reported synesthetes.

Finally we explored how these two types of test (consistency and speeded congruency) work in tandem in their assessments of synesthesia. If we take self-reported synesthetes as a single group (i.e., collapsing genuine synesthetes and malingerers) there was a significant inverse correlation between the consistency colour-distance score, and the speeded congruency accuracy score (*r*(122) = −.71, *p* < .001; see Fig. 5). Remembering that low colour-distance scores and high accuracy scores are both indicative of synesthesia, this inverse correlation shows that those who performed like synesthetes in the first sub-test were also more likely to perform like synesthetes in the second. However, when we calculate this correlation for genuine synesthetes and malingerers separately, it becomes apparent that the effect comes from the malingerer group only. The correlation between consistency colour distance score and speeded-congruency accuracy score for genuine synesthetes is non-significant (*r*(32) = −.07, *p* = .35) whereas for the malingerers, the correlation is highly significant (*r*(88) = −.73, *p* < .0001).

Finally, there was no significant correlation between consistency colour-distance score and reaction time (*r*(122) = −.05, *p* = .59), nor for accuracy score and reaction time (*r*(122) = −.11, *p* = .22), and this also remained the case when correlations for the synesthete and malingerer groups were calculated separately (synesthete consistency-RT = *r*(32) = .006, *p* = .49; malinger consistency-RT = *r*(88) = .014, *p* = .45) (synesthete accuracy-RT = *r*(32) = −.18, *p* = .14; malinger accuracy-RT = *r*(88) = −.09, *p* = .19).^5^

### 3.1 Prevalence comparison between studies: Comparing short versus long-term testing

The key aim of this study was to compare the prevalence of grapheme-colour synesthesia generated by the Synesthesia Battery (a single-session test) to a more conventional method based on long term (rather than single session) testing (Simner, Mulvenna et al., 2006). Using the Synesthesia Battery we found the prevalence of grapheme-colour synesthesia in the general population to be 1.2% for those with coloured letters *or* digits, compared to the previous estimate of 2% in conventional longer-term testing (Simner, Mulvenna et al., 2006). We can now evaluate that these two estimates are not significantly different (chi square = 2.1; *df* = 1; *p* = .14). However, we also calculated prevalence for synesthetes with both coloured letters *and* digits, finding a value here of 0.5%, and this is significantly less than the 1.4% found in conventional longer term retesting (Simner, Mulvenna et al., 2006; chi square = 5.6; *df* = 1; *p* = .02).

## 4 Discussion

In our study, we reproduced elements of the Synesthesia Battery (Eagleman et al., 2007) which is a single-session online test for grapheme-colour synesthesia. We used this method to estimate the prevalence of grapheme-colour synesthesia in a very large randomly recruited sample – indeed the largest sample for this purpose to date. We found the prevalence of grapheme-colour synesthesia in the general population to be 1.2% for those with coloured letters *or* digits, compared to the previous estimate of 2% (Simner, Mulvenna et al., 2006), a difference which is not significantly different. However, we also calculated prevalence of synesthetes with both coloured letters *and* digits, finding a value here of 0.5%, and this is significantly less than the 1.4% found in conventional longer term retesting (Simner, Mulvenna et al., 2006). Hence, the Synesthesia Battery numerically under-estimated the prevalence of those with coloured letters and digits, compared to longer retesting methods and we explore possible reasons for this below.

One explanation for an under-estimation of prevalence in the Synesthesia Battery might stem from the way graphemes are presented to those who report more than one variant: letters and digits are presented together, randomly ordered within the same consistency test. In the longer term retesting used as the baseline here however, letters and digits were always presented in separate blocks (Simner, Mulvenna et al., 2006). It may therefore be that synesthetes are susceptible to interference when selecting colours for graphemes, and we raise this possibility for future studies to consider. A second possible explanation for the lower prevalence found here is that immediate retests might be inherently more conservative, and this might be suggested by the fact that prevalence estimates here were numerically lower across the board. Immediate retesting might be conservative because the scores of *non-synaethetes* are likely to be better across shorter (versus longer) time intervals. In other words, non-synesthetes in an immediate retest (e.g., over 10 min) should score higher than in a delayed retest (e.g., over several weeks) even though synesthetes’ scores, in comparison, would be likely to remain relatively unchanged. This would raise the control baseline for single session testing, and therefore allow a smaller range of synesthetes to be identified as significantly more consistent. If this is correct, we conclude that the threshold at which synesthetes are identified (currently around the score of 1) might be usefully set marginally higher in the Synesthesia Battery of Eagleman et al. (2007).

One recent study has also speculated that the threshold in the Synesthesia Battery perhaps should be raised. Rothen et al. (2013) have recently argued that for the combination of RGB colour space and city block distance used by Eagleman et al. (2007) – and also in this study – a higher cut-off may indeed be more appropriate. In order to maximise sensitivity and specificity for this colour space/distance combination, they proposed a revised threshold within the existing Synesthesia Battery at <1.43 for synesthetes (rather than <1). In our own study, if we recalculate prevalence in our sample using Rothen et al’s suggested cut-off score of 1.43, our prevalence estimates are no longer significantly lower than expected. With this revised threshold, we now find 69 genuine synesthetes with coloured letters OR digits (2.4%; compared to 2% in Simner, Mulvenna et al., 2006; chi square = 0.3; *df* = 1; *p* = .57) and 31 synesthetes with coloured letters AND digits (1.1%; compared to 1.4% in Simner, Mulvenna et al., 2006; chi square = 0.367; *df* = 1; *p* = .5). In other words, our results are more in line with more conventional longer term evaluations of synesthesia when the threshold is shifted upwards according to the proposal of Rothen et al. (2013).^6^ Finally, we point out that Rothen and colleagues also suggest that using alternative colour models (CIELUV and CIELAB) and Euclidean distance provide the best combination of sensitivity and specificity in distinguishing between synesthetes and non-synesthetes. However that change would be beyond the scope of this paper, where we are simply evaluating the Eagleman data output as it stands.

Our discussion above of where to set the cut-off threshold for consistency in the Synesthesia Battery might be considered part of a more fundamental concern in synesthesia research. In all kinds of consistency tests for synesthesia – and particularly obvious here – participants are divided into two groups by their score on what is in fact an incremental continuum of possible scores. (Using this test, participants can, in theory, score any value between 0 and ~4, even though the cut-off is conventionally placed at the fixed value of 1). It seems clear that someone who scores 1.05 on the consistency test is “more synesthetic” than someone scoring 2.48, yet according to the cut-off of <1, both would be considered non-synesthetes. Nonetheless, it is a particular strength of the Synesthesia Battery that researchers are free to consider this score in its own right, rather than for categorical groupings alone.

The second part of Eagleman et al’s test involved speeded congruency task in which graphemes are presented either in the same colour previously selected (congruent) or a different colour (incongruent). Subjects must indicate whether the colour they saw matched their earlier choice, and Eagleman et al. (2007) report significant differences in speed and accuracy between a pre-selected group of 15 self-referred synesthetes, and a non-synesthete control group. In our current study, when our randomly-sampled respondents were divided into genuine synesthetes versus malingerers (i.e., around the threshold score of 1), genuine synesthetes were again significantly more accurate than malingerers. These data suggest that accuracy scores can not only distinguish between self-referred synesthetes and non-synesthetes (as in Eagleman et al., 2007) but is also subtle enough to distinguish between groups of genuine synesthetes and those who claim to be so, but do not pass a conventional consistency test.

Furthermore, considering all self-reported synesthetes irrespective of their consistency, there was a significant inverse correlation between consistency and accuracy, indicating that more consistent (i.e., more ‘genuine’) synesthetes were also more accurate. However, when this correlation was calculated separately for the each group (genuine synesthetes and malingers) it became apparent that the effect was driven by the malingerers only. We suggest this is because genuine synesthetes score highly on the accuracy test, irrespective of what consistency score they achieve. In other words, a synesthete obtaining a consistency score of, say, 0.99 is likely to be highly accurate on the speeded-congruency test – as accurate as a synesthete scoring 0.4 on the consistency test. In contrast, a malingerer obtaining a consistency score of 1.5 is more likely to be more accurate on the speeded-congruency test than a malingerer scoring 3.5 for consistency.

The speed accuracy congruency test presented here showed one notable difference in results compared to Eagleman et al. (2007). Our own findings were that genuine synesthetes, although more accurate than malingerers, were no faster. Eagleman and colleagues found genuine synesthetes to be more accurate *and* faster than controls. This difference to Eagleman et al. (2007) may stem from differences in our control populations: Eagleman et al. (2007) compared synesthetes to self-declared non-synesthetes, while we compared to ‘malingerer’ individuals claiming to have synesthesia. It is possible that some portion of our controls were in fact synesthetic in some way, albeit with lower consistency, and perhaps this is why our genuine synesthetes did not differ from them in their RTs (see above and Simner, 2012 for discussion). Alternatively, our lack of difference in RTs may be the result of the unanticipated variations we introduced in our version of this test. Our method of selecting congruent and incongruent graphemes and the timing of grapheme presentation for this part of the test differed slightly from Eagleman et al.’s original approach (see Section 2 for a full explanation). Our own version may have raised the difficulty of the task (e.g., because graphemes were on-screen for a shorter time on average) and our RTs were certainly longer and hence potentially more noisy. There is no way to distinguish between these two hypotheses in the current study and so we leave this as an open question for future studies to address.

We have evaluated whether single session tests of consistency are effective at identifying synesthetes, compared to established longer retesting methods. One previous study has also suggested that single session testing may indeed be valid. Simner, Mulvenna et al. (2006) established the prevalence of synesthesia both with long term testing (which we used here as our key comparison) but also by screening additional 1190 people in a single session. Their method was more basic than that of Eagleman et al. (2007) in that colour choices were made from a palette of just 13 colours, and only absolute matches contributed to consistency scores. Nonetheless, this again produced roughly comparable prevalence estimates as longer term testing (1.1% prevalence for coloured letters and digits). Taken together with the current study, we therefore suggest single session tests of consistency for synesthetic associations do appear to provide an appropriate method by which to identify synesthetes. The widely available nature of the Synesthesia Battery through its open-access online interface makes it a particularly appealing version of this test, as does its comprehensive colour palette, and its ability to give a calibrated estimate (i.e., continuous consistency score) for synesthesia status. Although researchers will want to consider carefully the question of whether consistency testing can reliably identify every type of synesthesia, or indeed every type of synesthete (see Simner, 2012 for discussion), it is clear from our current study that the Synesthesia Battery provides a suitable tool for evaluating synesthetes along this dimension.

## Footnotes

1 Early estimates of prevalence in the science literature had varied widely, at least partly because researchers were focussing on different sub-types or using different definitional criteria (Ramachandran & Hubbard, 2001). Even in studies that aimed to report the prevalence of all forms, estimates range from 1 in 4 (Calkins, 1895; Domino, 1989; Uhlich, 1957), to 1 in 10 (Rose, 1909), 1 in 20 (Galton, 1883), 1 in 200 (Ramachandran & Hubbard, 2001), 1 in 2000 (Baron-Cohen et al., 1996), and 1 in 25 000–100 000 (Cytowic, 1993, 1997). A number of these studies were simply ‘best guesses’, while others failed to use objective tests to verify synesthetic reports (e.g., Calkins, 1895; Domino, 1989; Rose, 1909; Uhlich, 1957). Others still did use robust consistency methods to verify synesthesia, but were subject to a recruitment bias: their subjects were recruited in response to adverts in newspapers, leaving open the possibility that low prevalence rates (e.g., 0.05–1% of the population; Barnett, Finucane, Asher, Bargary, Corvin, Newell, & Mitchell, 2008; Baron-Cohen et al., 1996) were due simply to poor rates of responding (see Baron-Cohen et al., 1996 for discussion). The estimate we use here (from Simner, Mulvenna et al., 2006) is a particularly robust estimate since it avoided the problems of self-referral by individually questioning every member of a participant pool, and using an objective test of genuineness (consistency over long-term retest).

2 On completing our study we discovered minor methodological differences to Eagleman et al. (2007) in how congruent and incongruently coloured graphemes were selected and presented. In our study here, the first presentation of graphemes included 50% that were randomly selected to be presented in in their congruent colour, taken from the participant’s 1st selection in the prior task. For the second presentation, 50% of graphemes were again randomly selected to be congruently coloured, this time from the participant’s 2nd selection from the previous task. The incongruent colours were randomly selected in RGB space, and were not taken from the participants’ earlier palette. In Eagleman et al. (2007), each grapheme was presented twice in a fully randomised order – once congruently (always from the 1st selection given previously) and once incongruently. The incongruent colour was chosen from the participant’s earlier palette of colours on the consistency test and verified to be sufficiently distant in RGB space from the congruent colour. The details of this were not given in the Eagleman paper. Furthermore, in our own study a response press advanced the display onto the next grapheme, while in Eagleman et al. (2007) the grapheme was displayed for a constant 1s followed by a fixed inter-stimulus interval. These differences mean we can no longer directly compare our data in this section to that from Eagleman et al. (2007). Instead, our investigation here will serve to determine whether this *type* of study (i.e., a speeded congruency task) can be validated on our new participant population and how responses in this type of test might relate to the consistency scores provided by the Synesthesia Battery. Finally we point out that our central aim – to assess the consistency portion of the Synesthesia Battery – is unaffected.

3 We use this term only for clarity but are in fact agnostic about the nature of these individuals. The prevailing view is that these are likely to be non-synesthetes falsely reporting synesthesia and therefore failing the objective consistency test. However, we leave open the possibility that some genuine synesthetes may not in fact be consistent over time, and refer to the reader to Simner, Mulvenna et al. (2006), Simner (2012), Eagleman (2012) and Cohen Kadosh and Terhune (2012) for further discussion on this matter.

4 We analysed both correct and incorrect trails was because our aim in this paper was to replicate Eagleman et al.’s (2007) methods as faithfully as possible. However, when analysing reaction times in Stroop-like behavioural tasks, convention typically dictates that only correct trials are included in the analysis. When only correct trials are analysed, the result does not change: there is no significant difference in reaction time between genuine synesthetes and malingerers (*p* = .95), which – like before – remains contrary to Eagleman et al.’s original finding.

5 An anonymous reviewer has asked us to comment on the age of our subjects. The age range of all participants was 16–90 years. No genuine synesthetes were found in the age range 52–90. Four participants in this age range reported having synesthesia but scored >1 in the colour distance consistency test, so were classified as malingerers.

6 If we repeat our earlier analyses from the speeded-congruency test (looking at differences in mean accuracy score and reaction time) grouping synesthetes and non-synesthetes according to the revised threshold of <1.43, neither result changes. Synesthetes remain significantly more accurate than non-synesthetes (82.8% (SD = 10.3) versus 63.7% (SD = 14.3); (*t*(95.27) = 8.32, *p* < .0001, *d* = 1.53). There remains no significant difference in reaction times between synesthetes and non-synesthetes (1.71 s (SD = 0.56) versus 1.58 s (SD = 0.81); (*t*(92.04) = 0.97, *p* < .17, *d* = 0.19).
